# Anti-granulocyte macrophage colony-stimulating factor autoantibodies: A rising cause of infectious diseases

**DOI:** 10.1371/journal.ppat.1014116

**Published:** 2026-04-20

**Authors:** Shang-Yu Wang, Yu-Fang Lo, Yu-Huan Tsai, Cheng-Lung Ku

**Affiliations:** 1 Laboratory of Human Immunology and Infectious Diseases, Institute of Immunology and Translational Medicine, Chang Gung University, Taoyuan, Taiwan; 2 Division of General Surgery, Chang Gung Memorial Hospital, Taoyuan, Taiwan; 3 School of Medicine, College of Medicine, Chang Gung University, Taoyuan, Taiwan; 4 Center for Molecular and Clinical and Immunology, Chang Gung University, Taoyuan, Taiwan; 5 Laboratory of Host–Microbe Interactions and Cell Dynamics, Department of Medical Biotechnology and Laboratory Science, College of Medicine, Chang Gung University, Taoyuan, Taiwan; 6 Graduate Institute of Biomedical Sciences, College of Medicine, Chang Gung University, Taoyuan, Taiwan; 7 Division of Infectious Diseases, Department of Pediatrics, Chang Gung Memorial Hospital, Taoyuan, Taiwan; 8 Center for Drug Research and Development, College of Human Ecology, Chang Gung University of Science and Technology, Taoyuan, Taiwan; McGill University, CANADA

## Anti-cytokine autoantibody as the phenocopy of inborn error of immunity

Autoantibodies are produced even in individuals with normal lymphocyte maturation. Current evidence suggests that approximately 55% to 75% of antibodies expressed during human B lymphocyte maturation are autoantibodies [1]. In later stages of maturation, B lymphocytes producing autoantibodies are typically inhibited through various mechanisms to maintain self-tolerance [1]. Nevertheless, autoantibodies can still be detected in healthy adults [2]. Cytokines, critical regulators of various immune and physiological functions, are widely applied in clinical settings. Apart from cases where cytokine therapies, such as interferon for hepatitis C, induce anti-cytokine autoantibodies, anti-cytokine autoantibodies are ubiquitous in individuals with various clinical manifestations [2]. The presence of anti-cytokine autoantibodies reflects a complex interplay between immune regulation and environmental triggers. These anti-cytokine autoantibodies, while often clinically silent, can disrupt immune homeostasis under specific conditions, leading to significant pathological consequences. Anti-granulocyte macrophage colony-stimulating factor autoantibodies (anti-GM-CSF-Abs) are one such example, detectable in approximately 3% of individuals [3]. In recent years, anti-cytokine autoantibodies have been increasingly recognized as a cause of immunodeficiency in severe infections [4]. Unlike inborn error of immunity (IEI), which are typically genetic origin and manifest in childhood [4], immunodeficiency due to anti-cytokine autoantibodies is usually acquired later in life and manifested the similar clinical phenotype as the cognate genetic defect, as considered as a phenocopy of these IEI. Now, anti-cytokine autoantibodies are also acknowledged as an etiology of this kind of immunodeficiency. In the following sections, we explore anti-GM-CSF-Abs, a type of anti-cytokine autoantibody, and their associated immunodeficiency phenotypes.

## Anti-GM-CSF-Ab blocks the physiological functions of GM-CSF

GM-CSF, initially identified as a hematopoietic growth factor, influences both hematopoietic progenitor cells and mature immune cells. GM-CSF is majorly produced by helper T cells, but also by other lymphocytes, macrophages, fibroblasts, endothelial cells, chondrocytes, and tumor cells in response to immunogenic stimuli, particularly inflammation-induced cytokines [5]. Under normal physiological conditions, GM-CSF levels are low, indicating its role as a conditionally induced cytokine. However, GM-CSF also has a constitutive function, notably maintaining alveolar surfactant homeostasis by supporting alveolar macrophage activity. This homeostatic role is particularly critical in lungs, where GM-CSF facilitates the clearance of surfactant lipids and proteins, preventing alveolar dysfunction, and sustained innate immunity against pulmonary pathogens.

The first recognized clinical phenotype associated with anti-GM-CSF-Abs, identified in the 1990s, was pulmonary alveolar proteinosis (PAP), a disease characterized by excessive accumulation of phospholipid-rich surfactant in the alveoli [6]. Anti-GM-CSF-Ab-related PAP, termed autoimmune PAP (aPAP), accounts for approximately 90% of PAP cases [7]. Autoimmune PAP phenocopies congenital PAP, which results from genetic mutations in the GM-CSF receptor. Both conditions share a pathogenesis involving impaired GM-CSF signaling. Normally, GM-CSF signaling enables alveolar macrophages to maintain surfactant homeostasis, preventing surfactant accumulation and respiratory impairment [8]. In aPAP, anti-GM-CSF-Abs neutralize GM-CSF, impairing alveolar macrophage maturation and function, ultimately resulting in disease manifestation [9]. Clinically, the diagnosis of aPAP frequently relies on bronchoalveolar lavage and high-resolution computed tomography (HRCT), revealing characteristic findings such as “crazy-paving” patterns and ground-glass opacities [10]. Serologically, the diagnosis involves identifying a critical threshold level of anti-GM-CSF-Abs known to correlate with disease development [9]. Current management for PAP relies primarily on palliative whole lung lavage (WLL) to physically remove accumulated surfactant. However, high recurrence rates and the invasive nature of WLL underscore a critical need for targeted interventions. Although inhaled recombinant GM-CSF has been utilized, its clinical efficacy remains inconsistent across patient cohorts [11].

## Anti-GM-CSF-Ab as a pathogenic factor for specific infectious diseases

**Fig 1:**
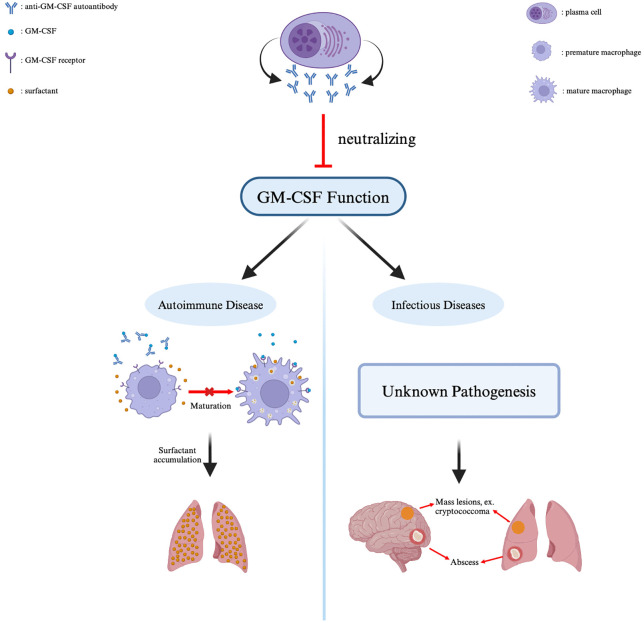
Clinical significance of anti-GM-CSF-Ab. Blockage of GM-CSF function by these autoantibodies results in clinical phenotypes, including autoimmune pulmonary alveolar proteinosis and specific infectious diseases. Figure created with BioRender.com. Created in BioRender. Chung, P. H. (2026) https://BioRender.com/fydf3kn.

**Fig 2:**
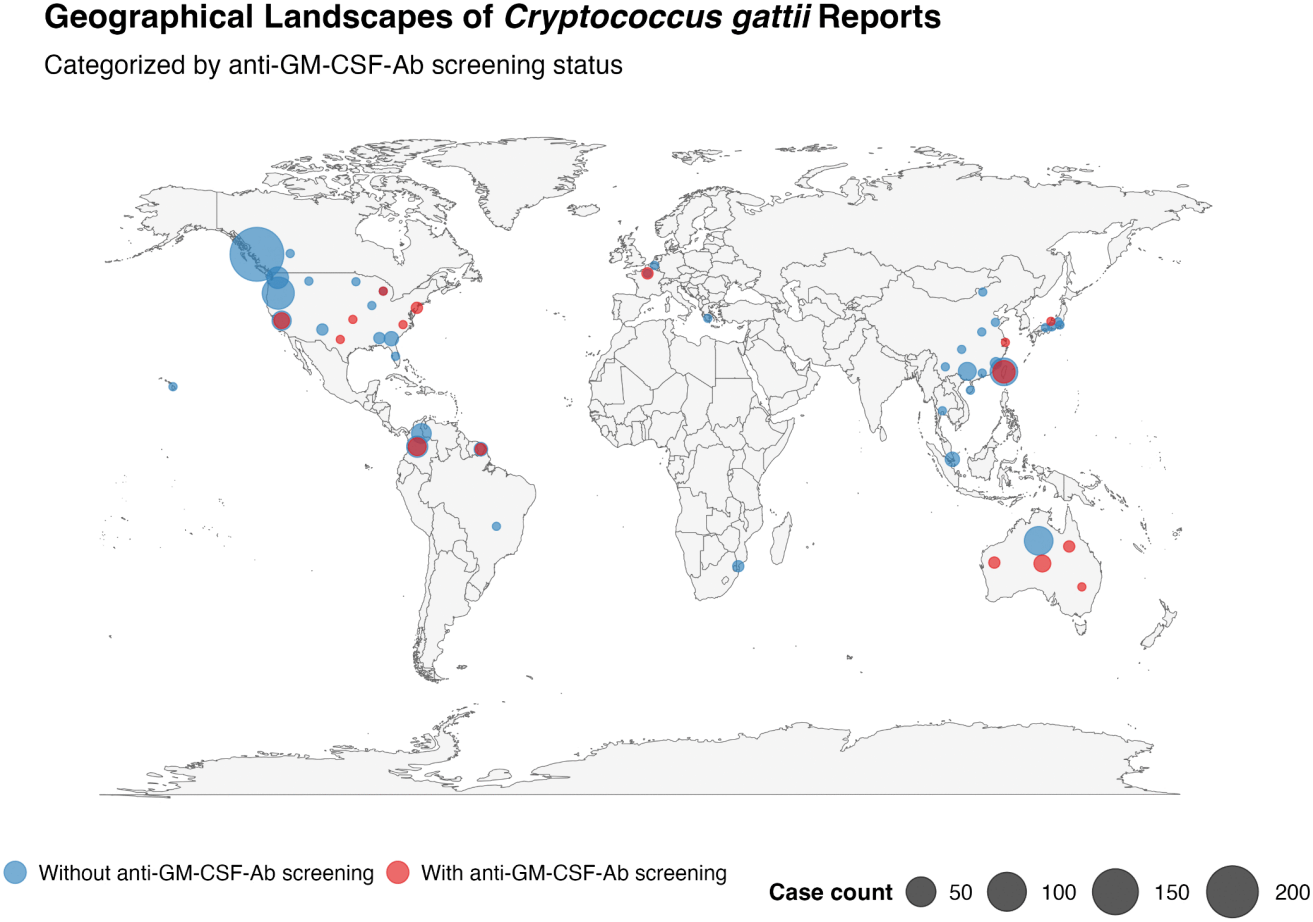
Global distribution of *Cryptococcus gattii* reports. Red and blue circles indicate study cohorts with and without anti-GM-CSF-Ab screening, respectively. Circle sizes are proportional to the total case count in each geographical region. This map was generated using R software with the “maps” and “ggplot2” packages. Base map data from the R ‘maps’ package (derived from CIA World DataBank II; public domain). Source Link: https://CRAN.R-project.org/package=maps.

In addition to the pathogenesis of aPAP, the anti-GM-CSF-Abs are recently unexpected linked to rare infectious diseases (Fig 1). While large, nationwide cohorts have reported PAP cases, infectious diseases associated with anti-GM-CSF-Abs are documented sporadically, and their pathogenesis is less understood [7]. The first association was reported by Rosen et al. in 2013, identifying anti-GM-CSF-Abs as a risk factor for cryptococcal meningitis [12]. In 2014, Saijo et al. demonstrated that anti-GM-CSF-Abs were specific to central nervous system (CNS) infections caused by *Cryptococcus gattii* (*C. gattii*) [13]. Subsequently, Kuo et al. reported a case series of patients with disseminated *C. gattii* cryptococcosis without aPAP [14]. Unlike *Cryptococcus neoformans* (*C. neoformans*), which primarily affects immunocompromised individuals, *C. gattii*-related cryptococcosis occurs in otherwise healthy individuals. Initially classified as *C. neoformans var. gattii*, *C. gattii* was formally established as a separate species in the early 2000s [15,16]. The *C. gattii* species complex comprises five genotypes (VGI ~ VGV), including *C. gattii sensu stricto* (VGI) and *C. deuterogattii* (VGII), the most common two genotypes in immunocompetent subjects [16]. The genotypes VGIII and VGIV mainly affect subjects with acquired immunodeficiency syndrome (AIDS) [17]. Regarding its environmental distribution, VGI, followed by VGII, is the most widely isolated genotype globally, with documented presence in Australia, North America, Europe (specifically the Netherlands and France), China, and Papua New Guinea [18,19]. In contrast, genotypes VGIII, VGIV, and VGV exhibit a more restricted geographical distribution [18]. There is a notable discrepancy between the environmental and clinical distributions of *C. gattii*. Many documented cases are linked to the presence of anti-GM-CSF-Abs, creating a publication bias that likely masks the actual clinical epidemiological landscape. After a thorough review, the geographical distribution of reported *C. gattii* infection is demonstrated in Fig 2, and the literature sources for the geographical analysis are summarized (S1 Data). While the Pacific Northwest remains, a well-recognized endemic region following its historical expansion, the identification of antibody-associated cases is predominantly concentrated in areas with active screening programs, such as Taiwan and Colombia. This suggests a significant diagnostic bias where the lack of routine autoantibody testing likely masks the true clinical prevalence of this occult immunodeficiency in other potentially endemic hotspots.

Although CNS involvement is most prevalent in reports, pulmonary infection is also a significant clinical manifestation. In North American outbreaks, *C. deuterogattii* (VGIIa, a molecular subtype of VGII) predominantly caused pulmonary infections, whereas in Australia and New Zealand, *C. gattii sensu stricto* frequently involved the CNS (74% to 86%) [20]. Unlike *C. neoformans* cryptococcosis, *C. gattii* infections are characterized by space-occupying lesions, which may mimic neoplastic diseases or pyogenic abscesses [20]. In an Asian cohort, patients with anti-GM-CSF-Abs and *C. gattii* cryptococcosis presented with cryptococcomas initially misdiagnosed as brain tumors, a feature absent in *C. neoformans* infections. The formation of cryptococcomas in *C. gattii* infections often complicates diagnosis, as these lesions may require biopsy or surgical interventions to differentiate from malignancies. Antifungal regimens for anti-GM-CSF-Ab cryptococcosis generally align with standard treatment used for cryptococcosis, and clinical outcomes for anti-GM-CSF-Abs-associated cryptococcosis are superior to those without autoantibodies, likely because these infections occur in otherwise healthy individuals [21]. Beyond *C. gattii* cryptococcosis, anti-GM-CSF-Abs are also associated with disseminated nocardiosis and invasive pulmonary aspergillosis, though these occur far less frequently [22–24].

## A decisive mechanism governs specific phenotype development

Available evidence suggests three major phenotypes associated with anti-GM-CSF-Abs: aPAP, *C. gattii* cryptococcosis, and nocardiosis. These phenotypes rarely coexist within the same individual, highlighting a striking selectivity in disease manifestation despite the shared underlying autoantibody presence. Although occasional case reports describe overlapping presentations, such as rare instances where patients might develop secondary infections alongside aPAP, real-world data and large cohort studies indicate minimal overlap [21,25]. A comprehensive Japanese cohort study involving 223 patients with aPAP, enrolled over six years in a national registry, and reported intercurrent infections in only about 5% of cases; notably, none of these infections involved *C. gattii* cryptococcosis or nocardiosis, underscoring the infrequency of such complications and the absence of phenotypic crossover in this large group [25]. Similarly, an in-depth analysis of the Taiwan National Health Insurance Research Database (NHIRD), which explored the epidemiology of cryptococcosis and PAP across a broad population, found no instances of overlap between PAP and cryptococcosis diagnoses [21]. While reports of overlapping phenotypes exist, publication bias may exaggerate their prevalence, as such atypical cases tend to prompt targeted testing for anti-GM-CSF-Abs and subsequentiy to be reported, potentially skewing perceptions of rarity [26]. The distinct phenotypic expression may be influenced by antibody titers, host genetic factors, or environmental exposures, which could modulate the immune response and determine disease specificity. Collectively, current evidence from these epidemiological and clinical investigations strongly supports the existence of a decisive immunological or environmental mechanism, possibly involving pathogen-specific interactions or subtle variations in autoantibody neutralization of GM-CSF signaling, underlying the selective manifestation of one particular phenotype over the others, emphasizing the need for further interdisciplinary research to unravel these determinants.

## Anti-GM-CSF-Abs require mechanistic study to explain their mysterious pathogen specificity and non-overlapping clinical phenotypes

Several unresolved questions remain regarding anti-GM-CSF autoantibodies-Abs. First, their associated infectious diseases show remarkable specificity, being almost exclusively linked to *C. gattii* and *Nocardia* species in CNS. The underlying reasons for this restricted pathogen spectrum and CNS tropism are unknown. Second, the three majorly described clinical phenotypes—aPAP, cryptococcosis, and nocardiosis—rarely overlap in the same patient, either synchronously or sequentially, suggesting distinct and non-redundant pathogenic mechanisms.

To address these questions, mechanistic studies are needed to dissect the role of GM-CSF in CNS and lung immunity. The documented role of GM-CSF in invasive pulmonary aspergillosis, provides valuable insights into the pathogenesis of anti-GM-CSF-Ab-associated cryptococcosis and nocardiosis. In *Aspergillus* models, GM-CSF acts as a critical licensing factor from lung epithelial cells that orchestrates the neutrophil oxidative burst and glycolytic reprogramming in monocytes [27,28]. We infer that a similar disruption of this pulmonary defense predisposes patients to *C. gattii* and *Nocardia*. Notably, unlike *Aspergillus*, which typically remains localized to the lungs, *C. gattii* and *Nocardia* exhibit a unique propensity for dissemination and CNS involvement, highlighting a distinct clinical phenotype despite a possibly shared underlying immune defect.

Addressing the remaining challenges in anti-GM-CSF-Ab-associated diseases will require multifaceted clinical and research strategies. Approaches such as animal infection models or advanced organoid systems could clarify how anti-GM-CSF-Ab shapes host defense against *C. gattii* and *Nocardia*. Besides, a more detailed characterization of the antibodies themselves, including their isotypes, affinities, epitope specificities, and effector functions, will be crucial to determine how they impair GM-CSF-mediated immune pathways. Such investigations may provide the basis for understanding disease specificity and heterogeneity and ultimately inform rational therapeutic strategies.

## Supporting information

S1 Data Literature sources and references for Fig 2. (DOCX)
