# Supplementary material for: Anti-granulocyte macrophage colony-stimulating factor autoantibodies: A rising cause of infectious diseases
Source: PLoS Pathog. 2026 Apr 20;22(4):e1014116. doi: 10.1371/journal.ppat.1014116 (PMC13094945; doi:10.1371/journal.ppat.1014116)
Supplement: S1 Data — (DOCX) [file ppat.1014116.s001.docx]

**S1 Data**

Supplementary References for Figure 2.

1. Dromer F, Ronin O, Dupont B. Isolation of Cryptococcus neoformans var. gattii from an Asian patient in France: evidence for dormant infection in healthy subjects. J Med Vet Mycol. 1992;30(5):395–7.

2. Peetermans W, Bobbaers H, Verhaegen J, Vandepitte J. Fluconazole-resistant Cryptococcus neoformans var gattii in an AIDS patient. Acta Clin Belg. 1993;48(6):405–9.

3. Lalloo D, Fisher D, Naraqi S, Laurenson I, Temu P, Sinha A, et al. Cryptococcal meningitis (C. neoformans var. gattii) leading to blindness in previously healthy Melanesian adults in Papua New Guinea. Q J Med. 1994;87(6):343–9.

4. Dengler R. Smoking and alcohol consumption in Trent, UK: an analysis of item non-response. J Epidemiol Community Health. 1996;50(6):687.

5. Dora JM, Kelbert S, Deutschendorf C, Cunha VS, Aquino VR, Santos RP, et al. Cutaneous cryptococccosis due to Cryptococcus gattii in immunocompetent hosts: case report and review. Mycopathologia. 2006;161(4):235–8.

6. Mansour A, Nakhla I, El Sherif M, Sultan YA, Frenck RW. Cryptococcus neoformans var. gattii meningitis in Egypt: a case report. East Mediterr Health J. 2006;12(1-2):241–4.

7. Bromilow J, Corcoran T. Cryptococcus gattii infection causing fulminant intracranial hypertension. Br J Anaesth. 2007;99(4):528–31.

8. Levy R, Pitout J, Long P, Gill MJ. Late presentation of Cryptococcus gattii meningitis in a traveller to Vancouver Island: A case report. Can J Infect Dis Med Microbiol. 2007;18(3):197–9.

9. Oliveira Fde M, Severo CB, Guazzelli LS, Severo LC. Cryptococcus gattii fungemia: report of a case with lung and brain lesions mimicking radiological features of malignancy. Rev Inst Med Trop Sao Paulo. 2007;49(4):263–5.

10. Galanis E. Epidemiology of Cryptococcus gattii, British Columbia, Canada, 1999–2007. Emerging Infectious Diseases. 2010.

11. Gutierrez EL, Valqui W, Vilchez L, Evangelista L, Crispin S, Tello M, et al. Cryptococcus gattii meningoencephalitis in an HIV-negative patient from the Peruvian Andes. Rev Soc Bras Med Trop. 2010;43(4):469–71.

12. Okamoto K, Hatakeyama S, Itoyama S, Nukui Y, Yoshino Y, Kitazawa T, et al. Cryptococcus gattii genotype VGIIa infection in man, Japan, 2007. Emerg Infect Dis. 2010;16(7):1155–7.

13. Pinto Junior VL, Pone MV, Pone SM, Campos JM, Garrido JR, de Barros AC, et al. Cryptococcus gattii molecular type VGII as agent of meningitis in a healthy child in Rio de Janeiro, Brazil: report of an autochthonous case. Rev Soc Bras Med Trop. 2010;43(6):746–8.

14. Steele KT, Thakur R, Nthobatsang R, Steenhoff AP, Bisson GP. In-hospital mortality of HIV-infected cryptococcal meningitis patients with C. gattii and C. neoformans infection in Gaborone, Botswana. Med Mycol. 2010;48(8):1112–5.

15. Harris JR, Lockhart SR, Debess E, Marsden-Haug N, Goldoft M, Wohrle R, et al. Cryptococcus gattii in the United States: clinical aspects of infection with an emerging pathogen. Clin Infect Dis. 2011;53(12):1188–95.

16. Leão CA, Ferreira-Paim K, Andrade-Silva L, Mora DJ, da Silva PR, Machado AS, et al. Primary cutaneous cryptococcosis caused by Cryptococcus gattii in an immunocompetent host. Med Mycol. 2011;49(4):352–5.

17. Lingegowda BP, Koh TH, Ong HS, Tan TT. Primary cutaneous cryptococcosis due to Cryptococcus gattii in Singapore. Singapore Med J. 2011;52(7):e160–2.

18. MacDougall L, Fyfe M, Romney M, Starr M, Galanis E. Risk factors for Cryptococcus gattii infection, British Columbia, Canada. Emerg Infect Dis. 2011;17(2):193–9.

19. Mistry N, Tan K, Shokravi M, Hoang L. Cryptococcus gattii infections with cutaneous involvement. J Cutan Med Surg. 2011;15(4):236–7.

20. Johannson KA, Huston SM, Mody CH, Davidson W. Cryptococcus gattii pneumonia. Cmaj. 2012;184(12):1387–90.

21. Dall Bello AG, Severo CB, Schio S, Severo LC. First reported case of cellulitis due to Cryptococcus gattii in lung transplantation recipient: a case report. Dermatology Online Journal. 2013;19(11).

22. Harris JR, Lockhart SR, Sondermeyer G, Vugia DJ, Crist MB, D'Angelo MT, et al. Cryptococcus gattii infections in multiple states outside the US Pacific Northwest. Emerg Infect Dis. 2013;19(10):1620–6.

23. Rosen LB, Freeman AF, Yang LM, Jutivorakool K, Olivier KN, Angkasekwinai N, et al. Anti-GM-CSF autoantibodies in patients with cryptococcal meningitis. J Immunol. 2013;190(8):3959–66.

24. Chan M, Lye D, Win MK, Chow A, Barkham T. Clinical and microbiological characteristics of cryptococcosis in Singapore: predominance of Cryptococcus neoformans compared with Cryptococcus gattii. Int J Infect Dis. 2014;26:110–5.

25. Inada T, Imamura H, Kawamoto M, Sekiya H, Imai Y, Tani S, et al. [Cryptococcus Neoformans Var. Gattii meningoencephalitis with cryptococcoma in an immunocompetent patient successfully treated by surgical resection]. No Shinkei Geka. 2014;42(2):123–7.

26. Leechawengwongs M, Milindankura S, Sathirapongsasuti K, Tangkoskul T, Punyagupta S. Primary cutaneous cryptococcosis caused by Cryptococcus gattii VGII in a tsunami survivor from Thailand. Med Mycol Case Rep. 2014;6:31–3.

27. Lizarazo J, Escandon P, Agudelo CI, Firacative C, Meyer W, Castaneda E. Retrospective study of the epidemiology and clinical manifestations of Cryptococcus gattii infections in Colombia from 1997-2011. PLoS Negl Trop Dis. 2014;8(11):e3272.

28. Nakashima K, Akamatsu H, Endo M, Kawamura I, Nakajima T, Takahashi T. Endobronchial cryptococcosis induced by Cryptococcus gattii mimicking metastatic lung cancer. Respirol Case Rep. 2014;2(3):108–10.

29. Saijo T, Chen J, Chen SC, Rosen LB, Yi J, Sorrell TC, et al. Anti-granulocyte-macrophage colony-stimulating factor autoantibodies are a risk factor for central nervous system infection by Cryptococcus gattii in otherwise immunocompetent patients. mBio. 2014;5(2):e00912–14.

30. Warren LR, Chandrasegaram MD, Neo EL, Dolan PM, Tan CP, Chen JW, et al. Large gas containing hepatic abscess following transarterial chemoembolization. ANZ J Surg. 2014;84(7-8):587–8.

31. Cicora F, Petroni J, Formosa P, Roberti J. A rare case of Cryptococcus gattii pneumonia in a renal transplant patient. Transpl Infect Dis. 2015;17(3):463–6.

32. Espinel-Ingroff A, Kidd SE. Current trends in the prevalence of Cryptococcus gattii in the United States and Canada. Infect Drug Resist. 2015;8:89–97.

33. Ho SW, Ang CL, Ding CS, Barkham T, Teoh LC. Necrotizing Fasciitis Caused by Cryptococcus gattii. Am J Orthop (Belle Mead NJ). 2015;44(12):E517–22.

34. Amburgy JW, Miller JH, Ditty BJ, Vande Lune P, Muhammad S, Fisher WS, 3rd. Cryptococcus gattii in an Immunocompetent Patient in the Southeastern United States. Case Rep Infect Dis. 2016;2016:8280915.

35. Deiss RG, Bolaris M, Wang A, Filler SG. Cryptococcus gattii Meningitis Complicated by Listeria monocytogenes Infection. Emerg Infect Dis. 2016;22(9):1669–71.

36. Nakao M, Muramatsu H, Takahashi T, Niwa S, Kagawa Y, Kurokawa R, et al. Cryptococcus gattii Genotype VGIIa Infection in an Immunocompetent Japanese Patient: A Case Report and Mini-review. Intern Med. 2016;55(20):3021–4.

37. Crum-Cianflone NF, Lam PV, Ross-Walker S, Rosen LB, Holland SM. Autoantibodies to Granulocyte-Macrophage Colony-Stimulating Factor Associated With Severe and Unusual Manifestations of Cryptococcus gattii Infections. Open Forum Infect Dis. 2017;4(4):ofx211.

38. Kuo CY, Wang SY, Shih HP, Tu KH, Huang WC, Ding JY, et al. Disseminated Cryptococcosis Due to Anti-Granulocyte-Macrophage Colony-Stimulating Factor Autoantibodies in the Absence of Pulmonary Alveolar Proteinosis. J Clin Immunol. 2017;37(2):143–52.

39. Maciel RA, Ferreira LS, Wirth F, Rosa PD, Aves M, Turra E, et al. Corticosteroids for the management of severe intracranial hypertension in meningoencephalitis caused by Cryptococcus gattii: A case report and review. J Mycol Med. 2017;27(1):109–12.

40. Panackal AA, Rosen LB, Uzel G, Davis MJ, Hu G, Adeyemo A, et al. Susceptibility to Cryptococcal Meningoencephalitis Associated With Idiopathic CD4(+) Lymphopenia and Secondary Germline or Acquired Defects. Open Forum Infect Dis. 2017;4(2):ofx082.

41. Ulett KB, Cockburn JW, Jeffree R, Woods ML. Cerebral cryptococcoma mimicking glioblastoma. BMJ Case Rep. 2017;2017.

42. Demir S, Chebib N, Thivolet-Bejui F, Cottin V. Pulmonary alveolar proteinosis following cryptococcal meningitis: a possible cause? BMJ Case Rep. 2018;2018.

43. Moritz D, Mena Lora A, Blumer B, Harrington AT. Recovery of Cryptococcus gattii from an Infected Ventriculo-Peritoneal Shunt, Illinois, USA. Emerg Infect Dis. 2018;24(7):1382–3.

44. Quah J, Low TB, Fong R. Disseminated Cryptococcus gattii infection preceding onset of pulmonary alveolar proteinosis. Respirol Case Rep. 2018;6(7):e00357.

45. Rodriguez-Goncer I, Bongomin F, Doran HM, Novak-Frazer L, Masania R, Moore CB, et al. A case of pulmonary cryptococcoma due to Cryptococcus gattii in the United Kingdom. Med Mycol Case Rep. 2018;21:23–5.

46. Zheng S, Tan TT, Chien JMF. Cryptococcus gattii Infection Presenting as an Aggressive Lung Mass. Mycopathologia. 2018;183(3):597–602.

47. Applen Clancey S, Ciccone EJ, Coelho MA, Davis J, Ding L, Betancourt R, et al. Cryptococcus deuterogattii VGIIa Infection Associated with Travel to the Pacific Northwest Outbreak Region in an Anti-Granulocyte-Macrophage Colony-Stimulating Factor Autoantibody-Positive Patient in the United States. mBio. 2019;10(1).

48. Hurtado JC, Castillo P, Fernandes F, Navarro M, Lovane L, Casas I, et al. Mortality due to Cryptococcus neoformans and Cryptococcus gattii in low-income settings: an autopsy study. Sci Rep. 2019;9(1):7493.

49. Jha MK, Mohanty A, Gupta P. Cryptococcus gattii meningitis in a diabetic adult in South India. J Family Med Prim Care. 2019;8(3):1253–6.

50. Stevenson B, Bundell C, Mulrennan S, McLean-Tooke A, Murray R, Brusch A. The significance of anti-granulocyte-macrophage colony-stimulating factor antibodies in cryptococcal infection: case series and review of antibody testing. Intern Med J. 2019;49(11):1446–50.

51. Andreou M, Cogliati M, Kolonitsiou F, Stroumpos C, Stamouli V, Ravazoula P, et al. Cryptococcus gattii infection in an immunocompetent host in Greece. Med Mycol Case Rep. 2020;27:1–3.

52. Huang C, Tsui CKM, Chen M, Pan K, Li X, Wang L, et al. Emerging Cryptococcus gattii species complex infections in Guangxi, southern China. PLoS Negl Trop Dis. 2020;14(8):e0008493.

53. Huynh J, Saddi V, Cooper P, Cheng AT, Meyer W, Chen S, et al. Unusual Presentation of Severe Endobronchial Obstruction Caused by Cryptococcus gattii in a Child. J Pediatric Infect Dis Soc. 2020;9(1):67–70.

54. Ishikawa D, Maruyama T, Nakamura T, Tsukagoshi S, Furuta N, Nagashima K, et al. Postpartum paradoxical expansion of cerebrocerebellar lesions associated with Cryptococcus gattii meningoencephalitis. Clin Neurol Neurosurg. 2020;196:105955.

55. Jin L, Cao JR, Xue XY, Wu H, Wang LF, Guo L, et al. Clinical and microbiological characteristics of Cryptococcus gattii isolated from 7 hospitals in China. BMC Microbiol. 2020;20(1):73.

56. Perrineau S, Guery R, Monnier D, Puel A, Lanternier F. Anti-GM-CSF Autoantibodies and Cryptococcus neoformans var. grubii CNS Vasculitis. J Clin Immunol. 2020;40(5):767–9.

57. Raghuraman N, Lopez JD, Carter EB, Stout MJ, Macones GA, Tuuli MG, et al. The effect of intrapartum oxygen supplementation on category II fetal monitoring. Am J Obstet Gynecol. 2020;223(6):905 e1– e7.

58. Baddley JW, Chen SC, Huisingh C, Benedict K, DeBess EE, Galanis E, et al. MSG07: An International Cohort Study Comparing Epidemiology and Outcomes of Patients With Cryptococcus neoformans or Cryptococcus gattii Infections. Clin Infect Dis. 2021;73(7):1133–41.

59. Gong S, Sivabalan P, Eisen DP. Hearing impairment as an unusual presenting sign of Cryptococcus gattii meningoencephalitis. BMJ Case Rep. 2021;14(4).

60. Nakao M, Fujita K, Arakawa S, Hayashi S, Tomita S, Sato H, et al. Treatment of Cryptococcus gattii Infection Using Voriconazole. Intern Med. 2021;60(22):3635–8.

61. Roman S, Millet C, Geris S, Manickam R, Mechineni A. Crazy vaping and crazy-paving, a case of E-Cigarette/Vaping-Associated Lung Injury (EVALI) with chest CT showing crazy-paving pattern. Radiol Case Rep. 2021;16(11):3208–12.

62. Thompson L, Porte L, Diaz V, Diaz MC, Solar S, Valenzuela P, et al. Cryptococcus bacillisporus (VGIII) Meningoencephalitis Acquired in Santa Cruz, Bolivia. J Fungi (Basel). 2021;7(1).

63. Viola GM, Malek AE, Rosen LB, DiNardo AR, Nishiguchi T, Okhuysen PC, et al. Disseminated cryptococcosis and anti-granulocyte-macrophage colony-stimulating factor autoantibodies: An underappreciated association. Mycoses. 2021;64(6):576–82.

64. Yang DH, England MR, Salvator H, Anjum S, Park YD, Marr KA, et al. Cryptococcus gattii Species Complex as an Opportunistic Pathogen: Underlying Medical Conditions Associated with the Infection. mBio. 2021;12(5):e0270821.

65. Goupil de Bouille J, Epelboin L, Henaff F, Migaud M, Abboud P, Blanchet D, et al. Case Report: Invasive Cryptococcosis in French Guiana: Immune and Genetic Investigation in Six Non-HIV Patients. Front Immunol. 2022;13:881352.

66. Kuo PH, Wu UI, Pan YH, Wang JT, Wang YC, Sun HY, et al. Neutralizing Anti-Granulocyte-Macrophage Colony-Stimulating Factor Autoantibodies in Patients With Central Nervous System and Localized Cryptococcosis: Longitudinal Follow-up and Literature Review. Clin Infect Dis. 2022;75(2):278–87.

67. Lee E, Miller C, Ataya A, Wang T. Opportunistic Infection Associated With Elevated GM-CSF Autoantibodies: A Case Series and Review of the Literature. Open Forum Infect Dis. 2022;9(5):ofac146.

68. Nielsen MC, Peterson JM, Shine B, Hornak JP, Esechie A, Bhatt S, et al. A Fatal Fungal Infection: Cryptococcus gattii (VGI) Meningitis in Texas. Open Forum Infect Dis. 2022;9(7):ofac236.

69. Wang SY, Lo YF, Shih HP, Ho MW, Yeh CF, Peng JJ, et al. Cryptococcus gattii Infection as the Major Clinical Manifestation in Patients with Autoantibodies Against Granulocyte-Macrophage Colony-Stimulating Factor. J Clin Immunol. 2022;42(8):1730–41.

70. Willett KL, Dalvin LA, Pritt BS, Fida M, Kasten MJ, Olsen TW. Cryptococcus gattii endogenous chorioretinitis. Am J Ophthalmol Case Rep. 2022;25:101283.

71. Arango-Franco CA, Migaud M, Ramírez-Sánchez IC, Arango-Bustamante K, Moncada-Vélez M, Rojas J, et al. Anti-GM-CSF Neutralizing Autoantibodies in Colombian Patients with Disseminated Cryptococcosis. J Clin Immunol. 2023;43(5):921–32.

72. Coussement J, Heath CH, Roberts MB, Lane RJ, Spelman T, Smibert OC, et al. Current Epidemiology and Clinical Features of Cryptococcus Infection in Patients Without Human Immunodeficiency Virus: A Multicenter Study in 46 Hospitals in Australia and New Zealand. Clin Infect Dis. 2023;77(7):976–86.

73. El-Atoum M, Hargarten JC, Park YD, Ssebambulidde K, Ding L, Chittiboina P, et al. Persistent neurological symptoms and elevated intracranial pressures in a previously healthy host with cryptococcal meningitis. BMC Infect Dis. 2023;23(1):407.

74. O'Hern JA, Koenen A, Janson S, Hajkowicz KM, Robertson IK, Kidd SE, et al. Epidemiology, management and outcomes of Cryptococcus gattii infections: A 22-year cohort. PLoS Negl Trop Dis. 2023;17(3):e0011162.

75. Wang A, Liu J, Liu J, Yang L, Yao S, Qin BE, et al. Reversible deafness and blindness in Cryptococcus gattii meningitis with a ventriculoperitoneal shunt: A case report and literature review. J Mycol Med. 2023;33(2):101357.

76. Arango-Franco CA, Rojas J, Firacative C, Migaud M, Agudelo CI, Franco JL, et al. Autoantibodies Neutralizing GM-CSF in HIV-Negative Colombian Patients Infected with Cryptococcus gattii and C. neoformans. J Clin Immunol. 2024;44(7):163.

77. Chen CT, Ho MW, Chung WH. Disseminated Cryptococcal Gattii infection in a patient with anti-granulocyte-macrophage colony-stimulating-factor autoantibody: a case report. BMC Infect Dis. 2024;24(1):1413.

78. Hamaguchi T, Uchida N, Fujita-Nakata M, Nakanishi M, Tsuchido Y, Nagao M, et al. Autochthonous Cryptococcus gattii genotype VGIIb infection in a Japanese patient with anti-granulocyte-macrophage colony-stimulating factor antibodies. J Infect Chemother. 2024;30(10):1069–75.

79. Pinchuk A, Geginat G, Rickerts V, Neyazi B, Stein KP, Mawrin C, et al. Late Relapse of Previous Pulmonary Cryptococcosis With Symptoms Resembling Cerebral Infarction: A Case Report. Case Rep Infect Dis. 2024;2024:3905985.

80. De La OVS, Adame-Garza JE, Gonzalez Plascencia M, Saenz Ramirez A, Castillo Cabrera RI, Gomez Figueroa IO. Rare Cutaneous Presentation of Disseminated Cryptococcus gattii Infection in an Immunocompetent Male: A Case Report. Cureus. 2025;17(12):e100079.

81. Galanis E, MacDougall L, Rose C, Chen SCA, Oltean HN, Cieslak PR, et al. Predictors of Cryptococcus gattii Clinical Presentation and Outcome: An International Study. Clin Infect Dis. 2025;80(5):1088–94.

82. Moreno-Soto AA, Cardenas-Golac RJ, Paredes-Obando MF, Vasquez-Ascate JJ, Sibina-Vela J, Ramirez-Garcia EA, et al. Cryptococcus gattii meningitis in an immunocompromised patient in a hospital in the Peruvian Amazon: case report. Rev Peru Med Exp Salud Publica. 2025;42(2):212–6.

83. Ye CX, Lin HQ, Wang ZR, Qiu GQ, Chen SD, Wu JS, et al. Pulmonary alveolar proteinosis complicated by pulmonary and intracranial cryptococcal infection: A case report. Medicine (Baltimore). 2025;104(30):e43536.

84. Zeng Y, Xiong T, Xiang W, Zhang J, Li L, Yuan W, et al. Two cases of refractory Cryptococcus gatti (C gatti) infection and literature review: Case report. Medicine (Baltimore). 2026;105(5):e46999.
